# Ultrasound measurement of laryngeal structures in the parasagittal plane for the prediction of difficult laryngoscopies in Chinese adults

**DOI:** 10.1186/s12871-020-01053-3

**Published:** 2020-06-02

**Authors:** Hongwei Ni, Chunming Guan, Guangbao He, Yang Bao, Dongping Shi, Yijun Zhu

**Affiliations:** 1 Department of Anesthesiology, Jiading District Central Hospital Affiliated Shanghai University of Medicine& Health Sciences, 1 Chengbei Road, Shanghai, 201800 P.R. China; 2 Mudanjiang Medical University, Mudanjiang, 157011 P.R. China

**Keywords:** Difficult airway, Endotracheal intubation, Ultrasound

## Abstract

**Background:**

Abnormal laryngeal structures are likely to be associated with a difficult laryngoscopy procedure. Currently, laryngeal structures can be measured by ultrasonography, however, little research has been performed on the potential role of ultrasound on the evaluation of a difficult laryngoscopy. The present study investigated the value of laryngeal structure measurements for predicting a difficult laryngoscopy.

**Objective:**

The main objective of this study was to explore the value of laryngeal structure measurements for predicting a difficult laryngoscopy.

**Methods:**

Two hundred and eleven adult patients (over 18 years old) were recruited to undergo elective surgery under general anesthesia via endotracheal intubation. Ultrasound was utilized to measure the distance between the skin and thyroid cartilage (DST), the distance between the thyroid cartilage and epiglottis (DTE), and the distance between the skin and epiglottis (DSE) in the parasagittal plane. These metrics were then investigated as predictors for classifying a laryngoscopy as difficult vs easy, as defined by the Cormack and Lehane grading scale.

**Results:**

Multivariate logistic regression showed that the DSE, but not DST or DTE, was significantly related to difficult laryngoscopies. Specifically, a DSE ≥ 2.36 cm predicted difficult laryngoscopies with a sensitivity and specificity of 0.818 (95% CI: 0.766–0.870) and 0.856 (95% CI: 0.809–0.904). Furthermore, when combining the best model constructed of other indicators (i.e. sex, body mass index, modified Mallampati test) to predict the difficult laryngoscopy, the AUC reached 93.28%.

**Conclusion:**

DSE is an independent predictor of a difficult laryngoscopy; a DSE cutoff value of 2.36 cm is a better predictor of a difficult laryngoscope than other ultrasound or physiological measurements for predicting a difficult laryngoscope. Nevertheless, it’s more valuable to apply the best model of this study, composed of various physiological measurements, for this prediction purpose.

## Background

A problematic laryngoscopy is the most direct cause of a difficult intubation. Difficult intubation is an emergency situation and an important procedural step during anesthesia, and its failure both threatens airway safety and can be a direct cause of morbidity and mortality for patients in emergency situations [1–3]. An accurate preoperative assessment therefore provides comprehensive planning and management for reducing the risk of unanticipated difficult airways. However, common clinical evaluation measurements (e.g. the modified Mallampati test, thyromental distance, inter-incisor distance, cervical mobility, sex, body mass index, etc.) have limited value with unsatisfactory sensitivities and specificities. Additionally, patient insubordination can further complicate test conditions and limit the practicality of such evaluation measurements as critical patients may not cooperate.

In recent years, ultrasound technology has been widely used in the field of airway imaging as a convenient and non-invasive method for the diagnosis and adjuvant therapy of lower airway conditions, such as pneumothorax [4], pulmonary embolism [5], atelectasis [6] and tracheostomy [7]. Upper airway imaging has also been explored by ultrasound [8, 9], as well as by ultrasound-guided nerve block, for clear endotracheal intubation [10] and laryngeal mask position determination [11]. Nevertheless, there have been few studies investigating the potential role of ultrasound for difficult airway prediction, but there are currently no accepted indicators or established methods for predicting a difficult airway.

The aim of this study was to assess whether measuring the laryngeal structures may be useful for the prediction of a difficult laryngoscopy procedure. Specifically, we selected three measurements from a variety of ultrasound metrics, including the distance between skin and epiglottis (DSE), the distance between the skin and thyroid cartilage (DST) and the distance between the thyroid cartilage and epiglottis (DTE) in the parasagittal plane. We chose these measurements because the laryngeal structures closer to the glottis are important visual markers when performing an intubation, and abnormal laryngeal structures can therefore interfere with our visual path to explore the glottis and intubate successfully. The parasagittal measurement avoids the effect of a high larynx and provides a clear view of the adjoining relationship with various larynx structures.

## Methods

The research performed here was approved by Ethics Committee of Jiading District Central Hospital Affiliated Shanghai University of Medicine & Health Sciences, and all patients provided written informed consent to participate. We recruited elective surgery patients (over 18 years old) who were administered tracheal intubations under general anesthesia. The prospective observational study was conducted in our hospital from May 2018 to October 2018. Patients with anatomical abnormalities of the head and neck, fractures of the maxillofacial or cervical bones, or airway trauma were excluded from this study.

### Airway assessment

DST: the distance between the skin and thyroid cartilage; DTE: the distance between the thyroid cartilage and epiglottis; DSE: the distance between the skin and epiglottis; MMT: The modified Mallampati test; IID: inter-incisor distance; TMD: thyromental distance; CM: cervical mobility; BMI: body mass index; CL: the Cormack and Lehane; NPV: negative predictive value; PPV: positive predictive value; OR: Odds ratio; CI: confidence interval; AUC: Area Under Curve;H: hyoid; TC: thyroid cartilage; E: epiglottis; A-M: junction of air and mucous membranes; SM: strap muscles.

Enrolled patients were subjected to a classical pre-anesthetic airway assessment by two trained nurse anesthetists in a waiting hall before being wheeled into the operating room. The modified Mallampati test (MMT), inter-incisor distance (IID), thyromental distance (TMD) and cervical mobility (CM) measurements were recorded. Basic demographic data, such as sex, age, body weight, height and body mass index (BMI), were also collected during the pre-anesthetic airway assessment.

The oropharyngeal status was evaluated using the MMT [12] by asking the patient to sit across from the observer at eye level, open his/her mouth as wide as possible, and to stick out his/her tongue without phonation. Class 1 and class 2 Mallampati scores generally indicate an easy intubation whereas class 3 and class 4 scores indicate a difficult intubation. The IID was defined as the distance from the upper to lower incisors on the midline, as measured when the patient’s mouth is open as wide as possible. The IID score is recorded as ≥4 cm or < 4 cm [13]. TMD was defined as the distance between the mentum and thyroid notch when the neck is fully extended. Patient were categorized into two groups based on the TMD being either ≤6 cm or > 6 cm [14]. A CM, or maximum range of motion from head to neck, of < 80° was regarded as abnormal.

### Ultrasound measurements

**Fig. 1:**
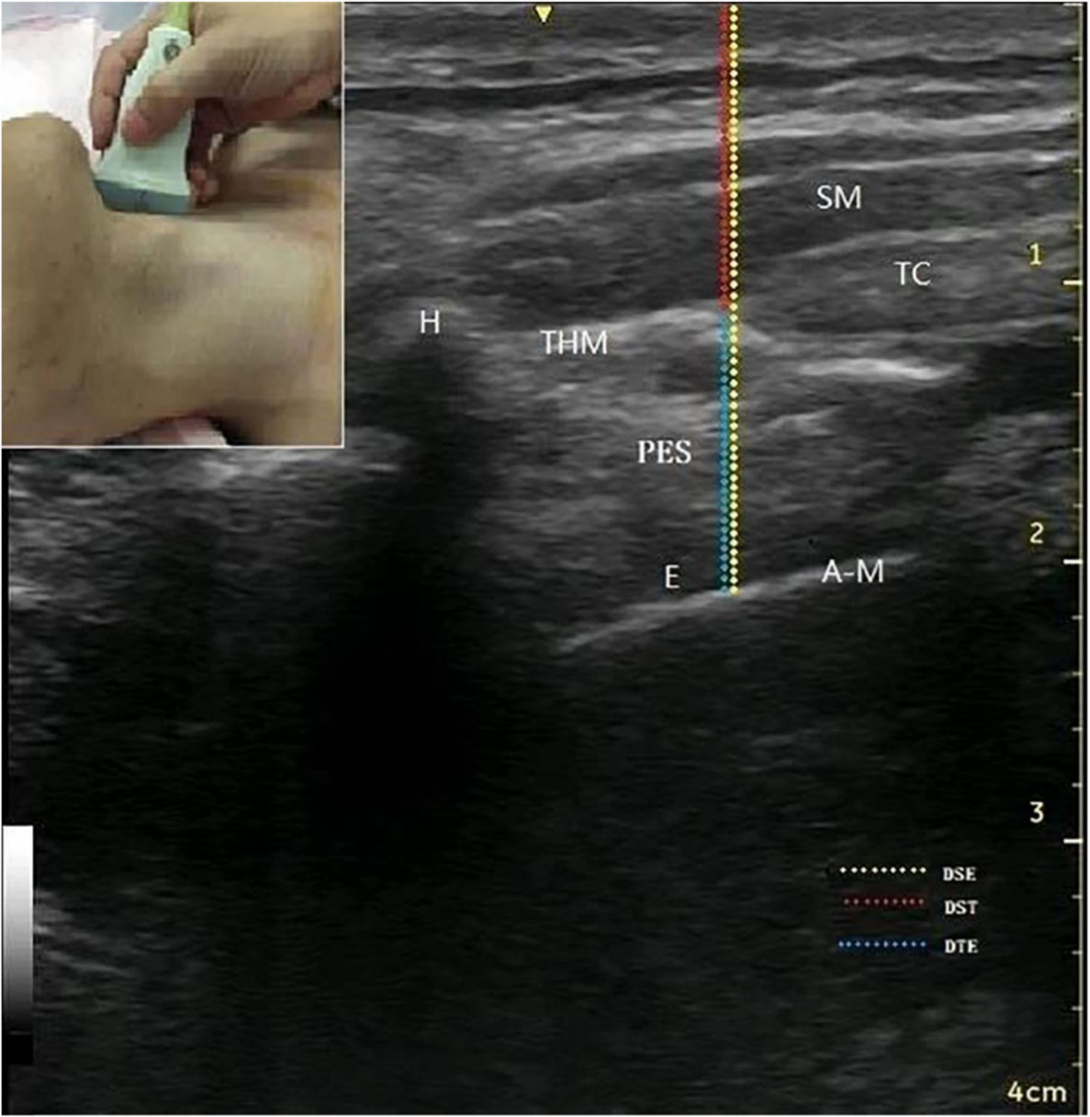
The parasagittal ultrasound view of laryngeal structures. H: hyoid; TC: thyroid cartilage; E: epiglottis; A-M: junction of air and mucous membranes; SM: strap muscles; DSE: distance between skin and epiglottis; DST: distance between skin and thyroid cartilage; DTE: distance between thyroid cartilage and epiglottis; PES: pre-epiglottis space

**Fig. 2:**
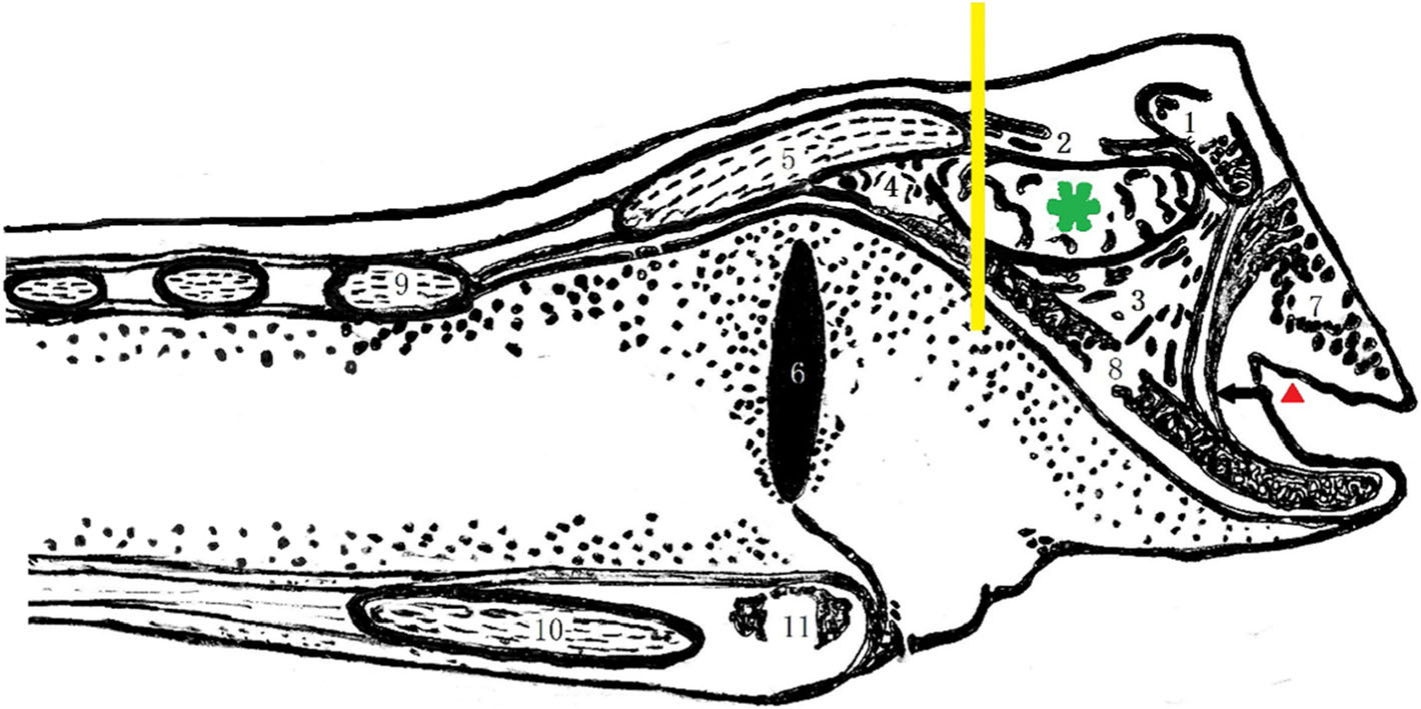
Schematic drawing of a sagittal section of the larynx. 1: hyoid bone; 2: thyrohyoid ligament;3: hyoepiglottic ligament; 4: thyroepiglottic ligament; 5: thyroid cartilage; 6: laryngeal ventricle; 7: root of tongue; 8: epiglottis; 9: cricoid arch; 10: cricoid lamina; 11: transverse arytenoid muscle; The yellow line indicates the measured section, the green asterisk the pre-epiglottic space containing pre-epiglottic fat pad, and the arrow the hypoepiglottic membrane. The red triangle indicates the epiglottic vallecula (where the tip of the laryngoscope blade is inserted)

After the pre-anesthetic airway assessment, patients were wheeled into the operating room where ultrasound measurements were performed by an experienced anesthesiologist who was blinded to the assessment results. In the supine position, the high-frequency linear (8-13 MHz) ultrasound probe (GE-Healthcare Venue 40 12 L-SC) was placed on the left or right (1 cm away from midline) side of the patient’s larynx for imaging in the parasagittal plane. In this orientation, the thyroid cartilage and hyoid bone were visible, and the interface between the air and the mucosa at the rear edge of epiglottis appeared as a hyperechoic line. The epiglottis can be confirmed by asking the patient to swallow slowly. At the level of the upper rim of the thyroid cartilage, the DST, DTE (including the thickness of the epiglottis itself) and DSE were measured, as shown as in Fig. 1 and Fig. 2. When the accuracy could not be determined, we chose the opposite measurement to define the accuracy.

### Laryngoscopy classification

After airway evaluations were completed, patients were monitored with intra-operative monitors that included blood pressure, pulse oximetry, end-tidal capnography and electrocardiography. Intravenous midazolam (2 mg), sufentanil (0.3 μg/kg), propofol (2 mg/kg) and cisatracurium (0.4 mg/kg) were administered 3 min before intubation. Another anesthesiologist who was unassociated with this study, with ≥5 years of experience, performed a laryngoscopy with a Macintosh blade and graded the airway using the Cormack and Lehane (CL) classification [15]. Specifically, the anesthesiologist who performed the endotracheal intubation recorded the first view of CL laryngoscopy classification without any external laryngeal maneuvers. Patients with CL grades of 3 or 4 were classified into the difficult laryngoscopy group, and those with CL grades of 1 or 2 were classified into the easy laryngoscopy group.

### Statistical analysis

The Stata 15.0 software package (StataCorp, College Station, TX) was used for all statistical analyses. We report categorical variables in the form of numbers (percentages) and compared the differences in such values between groups using a chi-square test. Continuous variables are represented as the mean ± standard deviation (SD), and a two-tailed t-test was used for comparison between groups. Twelve variables including Sex, Age, Weight, Height, BMI, TMD, CM, IID, MMT, DSE, DTE and DST were input into the model for analysis. Univariate logistic regression analysis was used to screen independent predictors for predicting a difficult laryngoscopy. The variance inflation factor (VIF) of each variable was detected to determine its multicollinearity. The non-condition stepwise logistic method was used to gradually remove variables using a backward elimination step with a threshold of *P* > 0.1 to determine the best model. The criteria for predicting a difficult laryngoscopy was determined by the Youden index. The sensitivity, specificity, negative predictive value, positive predictive value, KAPPA value, Jouden index and odds ratio, were used to evaluate the ability to predict a difficult laryngoscope for each variable and the best model. All comparisons were two-tailed and *P* <  0.05 was considered statistically significant.

Previous studies have demonstrated a about 20% incidence rate of a difficult laryngoscopy in surgical patients [13, 16, 17]. Therefore, a sample size of at least 185 patients would be required to demonstrate a difference in ultrasound evaluation between two groups with a type 1 error (α) of 5% and statistical power (1-β) of 95% (two-sided) using the PS program (Version 3.0).

## Results

**Fig. 3:**
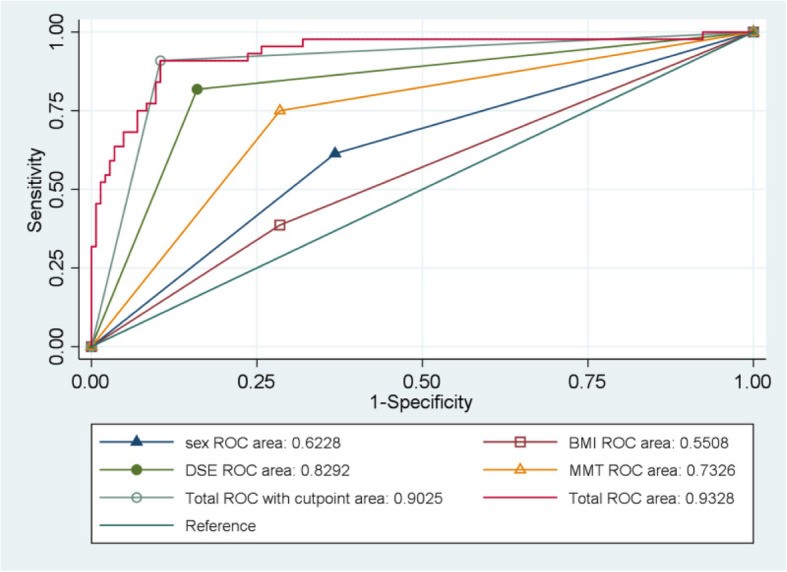
Receiver operator characteristic curve of the best model for predicting a difficult laryngoscopy, as well as that of the individual measurements. ROC: receiver operating characteristic; BMI: body mass index; MMT: modified mallampatia test; DSE: the distance from skin to epiglottis; OR: odds ratio

215 adult patients were recruited, and 4 patients were excluded before the final analysis (lack of data for ultrasound measurements). Of the 211 patients we successfully recruited for this study, 44 (20.85%) were diagnosed with a CL classification of level 3 or 4 (difficult laryngoscopy). In this study, laryngeal structures can be clearly seen in the parasagittal plane of ultrasound images, and the DST, DTE and DSE can be accurately measured. Descriptive data of the patients themselves and the airway assessment results are reported in Table 1. 27 male patients (61.36%) had a difficult laryngoscope in the difficult laryngoscope group, and suggest that men were more likely to experience a difficult laryngoscopy. Furthermore, there were significant differences in the MMT (*P* <  0.001), DSE (*P* <  0.001), DST (*P* <  0.001) and DTE (*P* <  0.001) between the easy and difficult laryngoscopy groups. Multivariate logistic regression showed that only the DSE was an independent predictor of laryngoscopy difficulties, but not DST and DTE. The optimal cutoff value of the DSE was 2.36 cm, as determined by the Youden index. Alternatively, the best model for predicting laryngoscopy difficulty included the four variables of sex, BMI, DSE and MMT, as shown in Table 2. There was no multicollinearity among the variables monitored by VIF. A comparison of the predictive power of the best model, as well as the individual components thereof (sex, BMI, DSE and MMT) was achieved by examining, for each case, the sensitivity, specificity, negative predictive value (NPV), positive predictive value (PPV), KAPPA value, Jouden index and Odds ratio (OR), as shown in Table 3. The DSE cutoff value of 2.36 cm alone predicted a difficult airway with an AUC of 0.8292 (95% CI: 0.774–0.901), a sensitivity of 0.818 (95% CI:0.766–0.870), a specificity of 0.856 (95% CI: 0.809–0.904), a PPV of 0.600 (95% CI: 0.534–0.666) and an NPV of 0.947 (95% CI: 0.917–0.977). It can be seen that the DSE is an effective predictor of difficult laryngoscopies and can be enhanced through the creation of a model that includes additional physiological indicators. By incorporating these additional factors, the AUC of a receiver operating characteristic curve reached 93.28%, as shown in Fig. 3.

**Table 1:** Descriptive data of the patients and the airway assessment results

Variable	Easy Laryngoscopy (***n*** = 167)	Difficult Laryngoscopy (***n*** = 44)	***p***-value
Sex			
Male	65 (38.92)	27 (61.36)	0.008
Female	102 (61.08)	17 (38.64)	
Age (y)	51.55 ± 14.60	54.48 ± 12.29	0.236
Weight (Kg)	63.23 ± 9.89	65.91 ± 12.29	0.131
Height (cm)	162.38 ± 7.55	164.86 ± 8.33	0.059
BMI (kg.cm^2^)	23.94 ± 3.16	24.21 ± 3.48	0.629
TMD < 4 cm	36 (21.56)	12 (27.27)	0.421
CM < 80°	38 (22.75)	6 (13.64)	0.981
IID < 4 cm	40 (23.95)	4 (9.09)	0.830
MMT ≥ III	11 (6.59)	33 (75.00)	< 0.001
DSE (cm)	2.05 ± 0.31	2.59 ± 0.41	< 0.001
DTE (cm)	0.99 ± 0.32	1.30 ± 0.39	< 0.001
DST (cm)	1.07 ± 0.28	1.28 ± 0.30	< 0.001

The results for continuous variables are represented by the mean ± standard deviation (SD). The categorical variables are in the form of numbers (percentages). *Abbreviations*: *BMI* Body mass index, *MMT* Modified mallampatia test, *TMD* Thyromental distance, *CM* Cervical mobility, *IID* Inter-incisor distance, *DSE* The distance between skin and epiglottis, *DST* The distance between skin and thyroid cartilage, *DTE* The distance between thyroid cartilage and epiglottis

**Table 2:** The best model selected by multivariate Logistic regression analysis

Variable	Odds Ratio	95% Confidence Interval	***P***-value
Sex	4.6232	1.5978 ~ 13.3773	0.005
BMI	0.5371	0.1762 ~ 1.6369	0.274
MMT	10.2082	3.3762 ~ 30.8647	< 0.001
DSE	38.7676	11.8096 ~ 127.2629	< 0.001

*Abbreviations*: *BMI* Body mass index, *MMT* Modified mallampatia test, *DSE* Distance between skin and epiglottis

**Table 3:** Comparison of sex, BMI, MMT, DSE and the best model for predicting a difficult laryngoscopy

Metric	Sex (Male)	BMI ≥ 25	MMT ≥ III	DSE > 2.36	Best Model
Sensitivity (95% CI)	0.614 (0.548–0.679)	0.386 (0.321–0.452)	0.750 (0.692–0.808)	0.818 (0.766–0.870)	0.909 (0.870–0.948)
Specificity (95% CI)	0.611(0.545–0.677)	0.731 (0.671–0.790)	0.713 (0.652–0.774)	0.856 (0.809–0.904)	0.904 (0.864–0.944)
PPV (95% CI)	0.294 (0.232–0.355)	0.274 (0.214–0.334)	0.407 (0.341–0.474)	0.600 (0.534–0.666)	0.714 (0.653–0.775)
NPV (95% CI)	0.857 (0.810–0.904)	0.819 (0.767–0.871)	0.915 (0.878–0.953)	0.947 (0.917–0.977)	0.974 (0.953–0.996)
Kappa	0.160 (0.043–0.278)	0.819 (0.767–0.871)	0.353 (0.230–0.476)	0.595 (0.463–0.727)	0.739 (0.606–0.873)
Youden	0.224	0.117	0.463	0.6745	0.8132
OR (95% CI)	2.492 (1.258–4.937)	1.707 (0.849–3.431)	7.437 (3.471–15.936)	26.813 (11.102–64.756)	

*Abbreviations*: *BMI* Body mass index, *MMT* Modified mallampatia test, *DSE* Distance from skin to epiglottis, *NPV* Negative predictive value, *PPV* Positive predictive value, *OR* Odds ratio, *CI* Confidence interval

## Discussion

Laryngeal structure measurements in the parasagittal plane are valuable for predicting laryngoscopy. While we found significant differences for each of these measurements between the easy and difficult laryngoscopy groups (Table 1), we found that the DSE was a particularly successful independent predictor of difficult laryngoscopies as evaluated by logistic regression. Furthermore, we found that various other physiological measurements played a role in optimizing the predictive power of difficult laryngoscopies. In addition to the DSE, the best predictive model included such parameters as sex, BMI, and MMT. While the utility of the MMT was expected, as it is a direct visual measurement of airway opening, we found it interesting that the other factors of sex and BMI also contributed to this optimal model, as we discuss in subsequent paragraphs. These factors suggest that simple physical tests can aid in predicting difficult laryngoscopies, however, more in-depth investigation into these parameters should be performed to draw concrete conclusions.

Ultrasound technology has recently been applied to the airway imaging field in recent years because it is a non-invasive and portable modality. Air and bone are considered to be the two major technical problems involved in ultrasound imaging, however, the artifacts induced by these substances can also be used as important diagnostic tools as long as their causes are understood. For example, ultrasound imaging has been previously explored for predicting difficult airways by detecting the artifactual air signal within the airway structure [8, 9]. With these foundations in place, clinical studies involving the prediction of a difficult airway are becoming more popular. Hui et al. [18] suggests that sublingual ultrasound can serve as a potential tool for predicting a difficult airway as a complementary measure to classical prediction methods. Along these lines, some studies suggest that the volume and thickness of the tongue can predict a difficult airway [19, 20], whereas other studies have implicated the neck circumference as a major predictor [21, 22]. More related to the current work, some studies have measured the anterior soft tissue thickness via ultrasound for predicting a difficult laryngoscopy [8, 23, 24], but these studies have yet not established a standard for which method is best.

In the current study, the ultrasound probe was placed along the parasagittal plane and mainly focused on the characteristics of the larynx structure itself. For men, the larynx is often much higher compared to women, and poor probe contact in these locations sometimes limited the visualization of the larynx structures and the median sagittal measurements. Prasad et al. [25] showed that the epiglottis can be seen in both the anterior transverse cervical plane and in the parasagittal plane; the epiglottis was more distinguishable between the hyoid bone and the thyroid cartilage in the parasagittal view. In current study, the epiglottis can be clearly seen in the parasagittal view, and the DST, DTE and DSE can be accurately and reliably measured (Fig. 1).

Pinto et al. [16] evaluated the use of the ultrasound-measured distance from the skin to epiglottis in the transverse plane and demonstrated that a cutoff value of 2.75 cm was effective for classifying easy vs difficult laryngoscopies. Falcetta et al. [26] also measured this same distance and found that a cutoff value of 2.54 cm was the most effective. Contrary to both of these previous works, we found that a DSE cutoff value of 2.36 cm was optimal, thus further presenting a level of variability that needs to be accounted for and/or corrected in future research. In the parasagittal plane, the DSE is the distance from the skin to the epiglottis between the hyoid bone and thyroid cartilage (Fig. 1 and Fig. 2), the ambiguity and movement of which may be why there is no accepted standard. According to the schematic drawing of the sagittal section of the larynx, we can clearly see the adjoining relationship of the various larynx structures. However, the region covered by the hypoepiglottic ligament can greatly change by lifting the epiglottis during intubation. We chose to measure at the upper rim of the thyroid cartilage, partly because of the bony markers in the location, and partly because the pre-epiglottal space is less affected by epiglottis movement during intubation. The ultrasound field of view in the parasagittal plane can visualize the landmarks such as the hyoid bone and thyroid cartilage which is located at the upper rim of thyroid cartilage. The measured distance is relatively stable, not including the hyoid epiglottic ligament area which is less affected by intubation. The parasagittal measurement avoids the effect of a high larynx and can clearly visualize the adjoining relationship with the various larynx structures. The DTE we measured incorporated the pre-epiglottal space composed of fat pads. To further analyze whether a difficult laryngoscopy is related to subcutaneous fat at the upper rim of the thyroid cartilage, we also measured the DST and DSE, which is in fact the sum of the DST and DTE (Fig. 1). Our results indicate that the DSE can serve as an independent predictor of a difficult laryngoscopy, but not the DST or DTE. This result suggests that if subcutaneous fat is thick at the level of the thyroid cartilage (DST) or there exists a large pre-epiglottal space (DTE), a difficult laryngoscopy cannot necessarily be predicted with confidence. However, when both of these features are present, the visual path to explore the glottis is noticeably obstructed and presents a scenario that can much more effectively predict the occurrence of a difficult laryngoscopy.

Current airway evaluation methods can be predictive but not definitive of difficult intubations, and a noticeable error rate exists because intubation difficulties are inherently subjective. The experience and ability of the anesthesiologist are likely the most important factors of a successful intubation. Different physicians subjectively graded the laryngoscope view and is a major source of uncertainty. Only by standardizing many variables, including the laryngoscopy equipment, experience of the anesthesiologists, the procedure for the first view of the glottis (used for Cormack-Lehane classification, without external laryngeal maneuvers during classification), can we reduce the possibility of bias and subjectivity introduced by the individual opinion of different anesthesiologists. Therefore, we chose the same anesthesiologist with ≥5 years of experience and used the first laryngoscopic view of the CL classification as a replacement indicator of difficult intubation. In the study, the incidence of difficult laryngoscopies at the first view was 20.85%, which was similar to other reports in literature [16, 17, 27, 28]. Various studies [22, 29] have shown that men are more likely to have difficulty with the laryngoscopy procedure, and our study also shows that men are at higher risk for a difficult laryngoscopy. When considering the subject’s BMI, we used the cut-off value of 25, which exactly is the definition of overweight set by the WHO [30]. Quinn et al.’s research shows that for every 1-point increase in BMI, there is a 7% increased risk of intubation failure. The modified Mallampati classification is a commonly used method of airway assessment in the clinic [31] and has been shown in previous studies to produce a wide range of sensitivity (42–81%) and specificity (66–84%) values for predicting a difficult laryngoscopy [32, 33]: In our study, the MMT displayed a sensitivity of 0.750 (95% CI: 0.692–0.808) and specificity of 0.713 (95% CI: 0.652–0.774), which are consistent with previous studies. Despite these promising results, of all ultrasound measurements collected in the current study, the DSE was the only one that was found to be a statistically significant independent indicator for predicting difficulty laryngoscopies, resulting in a sensitivity of 0.818 (95% CI: 0.766–0.870) and specificity of 0.856 (95% CI: 0.809–0.904). Nevertheless, by utilizing a “best” model, constructed with other indicators in addition to the DSE, to predict the difficult airway, the AUC reached 93.28%.

### Limitations

Firstly, only one ultrasound machine and one special experienced anesthesiologist performed the ultrasound airway evaluation and it was difficult for us to enroll all patients who met the inclusion criteria. Therefore, a very small number of patients were selected but did not receive a preoperative ultrasound evaluation. Therefore, it was difficult for the subjects to be randomly selected and a bias may have remained. Secondly, in two cases, the thyroid cartilage was obviously calcified, and the accurate measurement of the DST and DTE was limited (with no effect on the DSE measurements).

## Conclusions

Ultrasound is noninvasive, fast and reliable and does not require significant patient cooperation. Furthermore, laryngeal structure measurements in the parasagittal plane are valuable for predicting a difficult laryngoscopy. The DSE can be used to distinguish difficult and easy laryngoscopies; the DSE cutoff value of 2.36 cm resulted in a more powerful predictive value than other indicators for predicting a difficult laryngoscopy. Nevertheless, the combination of various parameters in a “best” model was the ideal case for predicting a difficult laryngoscopy in the current study. These results may provide further assistance in the clinical evaluation of difficult laryngoscopy.

## Supplementary information

**Additional file 1.**

## Data Availability

The datasets generated and analyzed during the current study are not publicly available due to un-obtaining permission from participants for the dataset. But are available from the corresponding author on reasonable request.

## Abbreviations

DST: The distance between the skin and thyroid cartilage
DTE: The distance between the thyroid cartilage and epiglottis
DSE: The distance between the skin and epiglottis
MMT: The modified Mallampati test
IID: Inter-incisor distance
TMD: Thyromental distance
CM: Cervical mobility
BMI: Body mass index
CL: The Cormack and Lehane
NPV: Negative predictive value
PPV: Positive predictive value
OR: Odds ratio
CI: Confidence interval
AUC: Area Under Curve
H: Hyoid
TC: Thyroid cartilage
E: Epiglottis
A-M: Junction of air and mucous membranes
SM: Strap muscles
