# Supplementary material for: Ultrasound measurement of laryngeal structures in the parasagittal plane for the prediction of difficult laryngoscopies in Chinese adults
Source: BMC Anesthesiol. 2020 Jun 2;20:134. doi: 10.1186/s12871-020-01053-3 (PMC7265219; doi:10.1186/s12871-020-01053-3)
Supplement: Supplementary file 1 — Additional file 1. [file 12871_2020_1053_MOESM1_ESM.docx]

Comparison of sex, BMI, MMT, DSE, Model 1 and Model 2 for predicting a difficult laryngoscopy

| **Metric** | **Sex (Male)** | **BMI ≥ 25** | **MMT ≥ III** | **DSE > 2.36** | **Model 1** | **Model 2** |
| --- | --- | --- | --- | --- | --- | --- |
| Sensitivity  (95% CI) | 0.614  (0.548-0.679) | 0.386  (0.321-0.452) | 0.750  (0.692-0.808) | 0.818  (0.766-0.870) | 0.750  (0.878-0.623) | 0.909  (0.870-0.948) |
| Specificity  (95% CI) | 0.611  (0.545-0.677) | 0.731  (0.671-0.790) | 0.713  (0.652-0.774) | 0.856  (0.809-0.904) | 0.743  (0.809-0.677) | 0.904  (0.864-0.944) |
| PPV  (95% CI) | 0.294  (0.232-0.355) | 0.274  (0.214-0.334) | 0.407  (0.341-0.474) | 0.600  (0.534-0.666) | 0.434  (0.546-0.323) | 0.714  (0.653-0.775) |
| NPV  (95% CI) | 0.857  (0.810-0.904) | 0.819  (0.767-0.871) | 0.915  (0.878-0.953) | 0.947  (0.917-0.977) | 0.919  (0.965-0.873) | 0.974  (0.953-0.996) |
| Kappa | 0.160  (0.043-0.278) | 0.819  (0.767-0.871) | 0.353  (0.230-0.476) | 0.595  (0.463-0.727) | 0.386  (0.263-0.514) | 0.739  (0.606-0.873) |
| Youden | 0.224 | 0.117 | 0.463 | 0.6745 | 0.493 | 0.8132 |
| OR  (95% CI) | 2.492 (1.258-4.937) | 1.707 (0.849-3.431) | 7.437 (3.471-15.936) | 26.813 (11.102-64.756) |  |  |

Abbreviations: BMI: body mass index; MMT: modified mallampatia test; DSE: distance from skin to epiglottis; NPV: negative predictive value; PPV: positive predictive value; OR: odds ratio; CI: confidence interval

Model 1 ：the combined physical parameters (sex, BMI and MMT without the DSE)

Model 2 : the combined physical parameters (sex, BMI , MMT and the DSE)

Comperation of Model 1 ROC area and Model 2 ROC area


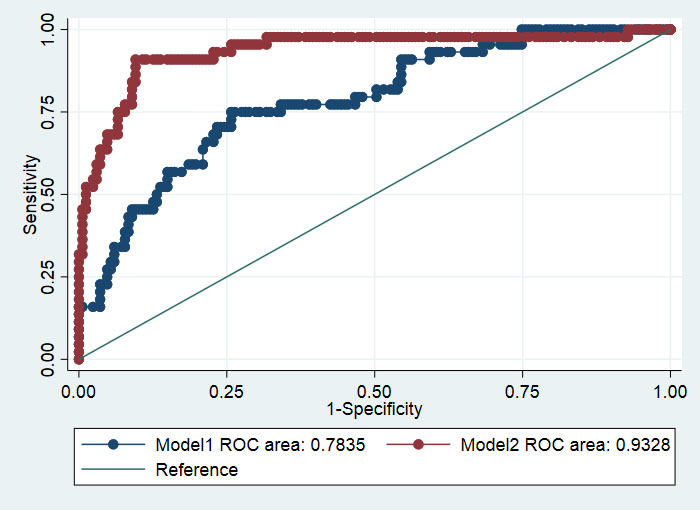


Receiver operator characteristic curves of Model 1 and Model 2 for predicting a difficult laryngoscopy. ROC: receiver operating characteristic.

Model 1：the combined physical parameters (sex, BMI and MMT without the DSE)

Model 2: the combined physical parameters (sex, BMI, MMT and the DSE)
